# Restaurant Food Allergy Practices — Six Selected Sites, United States, 2014

**DOI:** 10.15585/mmwr.mm6615a2

**Published:** 2017-04-21

**Authors:** Taylor J. Radke, Laura G. Brown, Brenda Faw, Nicole Hedeen, Bailey Matis, Priscela Perez, Brendalee Viveiros, Danny Ripley

**Affiliations:** ^1^Division of Emergency and Environmental Health Services, National Center for Environmental Health, CDC; ^2^California Environmental Health Specialists Network; ^3^Minnesota Department of Health; ^4^New York City Department of Health and Mental Hygiene; ^5^New York State Department of Health; ^6^Rhode Island Department of Health; ^7^Tennessee Department of Health.

Food allergies affect an estimated 15 million persons in the United States (*1*), and are responsible for approximately 30,000 emergency department visits and 150–200 deaths each year (*2*). Nearly half of reported fatal food allergy reactions over a 13-year period were caused by food from a restaurant or other food service establishment (*3*). To ascertain the prevalence of food allergy training, training topics, and practices related to food allergies, CDC’s Environmental Health Specialists Network (EHS-Net), a collaborative forum of federal agencies and state and local health departments with six sites, interviewed personnel at 278 restaurants. Fewer than half of the 277 restaurant managers (44.4%), 211 food workers (40.8%), and 156 servers (33.3%) interviewed reported receiving food allergy training. Among those who reported receiving training, topics commonly included the major food allergens and what to do if a customer has a food allergy. Although most restaurants had ingredient lists for at least some menu items, few had separate equipment or areas designated for the preparation of allergen-free food. Restaurants can reduce the risk for allergic reactions among patrons by providing food allergy training for personnel and ingredient lists for all menu items and by dedicating equipment and areas specifically for preparing allergen-free food.

Within each of the six EHS-Net sites (California, Minnesota, New York, New York City, Rhode Island, and Tennessee), data collectors chose a convenient geographic area, based on reasonable travel distance, in which to survey restaurants by telephone to determine their study eligibility and request participation. Within each geographic area, a random sample of restaurants was selected using statistical software. Restaurants were defined as facilities that prepare and serve food or beverages to customers and are not food carts, mobile food units, temporary food stands, or caterers, and are not located in supermarkets or institutions. Only restaurants with an English-speaking manager were eligible to be included in the study. Data collectors assessed approximately 50 restaurants in each of the sites. Data were collected during January 2014–February 2015.

After obtaining permission from the restaurant manager, data collectors conducted an on-site interview with a worker who had authority over the kitchen (manager), a worker who primarily prepared or cooked food (food worker), and a worker who primarily took orders or served food to customers (server). To increase participation and cooperation, data collectors asked the manager to select the English-speaking food worker and server to be interviewed. Data collectors interviewed all three personnel groups about food allergy training they had received while working at their current restaurant, including training topics covered (e.g., what are the major food allergens?). Managers also were asked whether the restaurant had ingredient lists for menu items accessible to the staff, customers, or both and whether special equipment or resources were dedicated to serving customers with food allergies (i.e., does this restaurant have a special area in the kitchen for making allergen-free food?).

Among the 1,307 restaurants contacted for participation in the study, 852 met the study eligibility criteria, and 278 (32.6%) of those agreed to participate; 58 restaurants were excluded because they did not have an English-speaking manager, and another 177 were excluded because they did not meet the restaurant definition for EHS-Net inclusion. Data on restaurant, manager, food worker, and server characteristics have been published previously (*4*). Among 277 managers, 123 (44.4%) reported that they had received training on food allergies while working at their respective restaurants (Table 1). Manager food allergy trainings most often covered how to prevent cross-contact (the inadvertent transfer of allergens from food, equipment, or surfaces containing an allergen to a food that does not contain the allergen) (96.7%); the major food allergens (milk, eggs, fish, shellfish, tree nuts, peanuts, wheat, and soybeans) (92.7%); and what to do if a customer has a food allergy (80.5%).

**TABLE 1:** Responses to questions regarding food allergy training reported by restaurant managers, food workers, and servers* — six Environmental Health Specialists Network sites,^Ϯ^ United States, 2014

Question	Managers (N = 277)	Food workers (N = 211)	Servers (N = 156)
	No. (%)	No. (%)	No. (%)
**Have you had training on food allergies while working at this restaurant?**			
Yes	123 (44.4)	86 (40.8)	52 (33.3)
No	152 (54.9)	123 (58.3)	103 (66.0)
Unsure	2 (0.7)	2 (1.0)	1 (0.6)
**Did your training cover^§^**			
How to prevent cross-contact from food allergens to other foods?	119 (96.7)	85 (98.8)	44 (84.6)
The most common, or major, food allergens?	114 (92.7)	74 (86.0)	45 (86.5)
What to do if a customer says they have a food allergy?	99 (80.5)	78 (90.7)	49 (94.2)
The menu items with food allergens in this restaurant?	85 (69.1)	66 (76.7)	41 (78.8)
The symptoms of an allergic reaction?	83 (67.5)	54 (62.8)	32 (61.5)
What to do if a customer has a bad food allergic reaction (e.g., trouble breathing)?	79 (64.2)	60 (69.8)	38 (73.1)

* Percentages might not sum to 100% because of rounding.

^Ϯ^ California, Minnesota, New York, New York City, Rhode Island, and Tennessee.

^§^ Denominators for percentages are the number of respondents in each group who said they had received training: 123 managers, 86 food workers, and 52 servers.

Among 211 food workers, 86 (40.8%) reported receiving food allergy training while working at their respective restaurants. Food worker food allergy trainings most often covered how to prevent cross-contact (98.8%), what to do if a customer has a food allergy (90.7%), and the major food allergens (86.0%).

Among 156 servers, 52 (33.3%) reported receiving food allergy training while working at their respective restaurants. Server food allergy trainings most often covered what to do if a customer has a food allergy (94.2%), the major food allergens (86.5%), and how to prevent cross-contact (84.6%). Across all three restaurant personnel groups, fewer participants reported that training covered menu items with food allergens (69.1% of managers, 76.7% of food workers, and 78.8% of servers), symptoms of an allergic reaction (67.5% of managers, 62.8% of food workers, and 61.5% of servers), and what to do if a customer has a bad allergic reaction (e.g., difficulty breathing) (64.2% of managers, 69.8% of food workers, and 73.1% of servers) (Table 1).

Among managers, 55.2% reported that their restaurants had ingredient lists or recipes for all or most menu items, 18.4% reported ingredient lists for some menu items, and 25.3% reported having no lists (Table 2). Among managers, 19.1% reported that their restaurants had a dedicated set of utensils or equipment for making allergen-free food (a meal free of the allergen to which a patron is allergic), and 78.0% reported no dedicated set of utensils or equipment. Few managers reported that their restaurant had a special area in the kitchen for preparing allergen-free food (7.6%), a special fryer for cooking allergen-free food (10.3%), or a special pick-up area for customers with food allergies (7.2%).

**TABLE 2:** Restaurant manager responses to questions regarding food allergy practices — six Environmental Health Specialists Network sites,* United States, 2014

Question (No. of respondents)	No. (%)
**Does this restaurant have lists or recipes with the ingredients for the food it makes? (277)**	
Yes for all or most menu items	153 (55.2)
Yes for some menu items	51 (18.4)
No	70 (25.3)
Unsure	3 (1.1)
**Does this restaurant have a special set of utensils or equipment for making allergen-free food? (277)**	
Yes	53 (19.1)
No	216 (78.0)
Unsure	8 (2.9)
**Does this restaurant have a special area in the kitchen for making allergen-free food? (277)**	
Yes	21 (7.6)
No	251 (90.6)
Unsure	5 (1.8)
**If there is a fryer in the restaurant, is there a special fryer for cooking allergen-free food? (214)**	
Yes	22 (10.3)
No	188 (87.8)
Unsure	4 (1.9)
**If there is a pick-up area in the restaurant, is there a special pick-up area for food for food allergic customers? (249)**	
Yes	18 (7.2)
No	228 (91.6)
Unsure	3 (1.2)

* California, Minnesota, New York, New York City, Rhode Island, and Tennessee.

## Discussion

The findings in this report suggest that there is considerable opportunity for restaurants to improve their practices to prevent allergic reactions among their patrons with food allergies. The 2013 Food and Drug Administration Food Code (*5*), which provides the basis for state and local codes that regulate retail food service, recommends that the person-in-charge (i.e., the manager) be knowledgeable about food allergies. Managers are also responsible for ensuring that employees are properly trained in food safety, including food allergy awareness. The organization Food Allergy Research & Education (FARE) encourages food allergy training for all new restaurant employees before they begin serving patrons, and periodic training updates for current staff members (*6*). However, the findings in this report indicate that employee training might not be occurring according to recommendations. Approximately half of surveyed restaurants did not provide food allergy training for their staffs, and the training provided often did not cover important information such as what to do if a customer has an allergic reaction (e.g., difficulty breathing). FARE guidance stresses the importance of staff members responding appropriately to allergic reactions (*6*).

Approximately one fourth of surveyed managers reported having no ingredient lists or recipes for menu items. Ingredient lists are important to help the staff determine which menu items contain common allergens. FARE recommends that a restaurant be able to supply, upon request, a list of ingredients for any menu item (*6*). 

Few surveyed restaurants had dedicated equipment for preparing allergen-free food. This is concerning because proteins from allergens can remain on equipment even after it is wiped clean, and dedicated equipment for making allergen-free food can reduce the risk for cross-contact. Dedicated equipment can be color-coded for quick identification, and designating areas in the kitchen for preparing allergen-free meals can further reduce the risk to patrons with food allergies. In addition, having a separate pick-up area can prevent problems such as delivering the wrong food to patrons, adding inappropriate garnishes, or exposing allergen-free meals to cross-contact with a food allergen (e.g., unclean hands, trays, or splashed food). Oils in deep fryers that are used to cook several different foods can contain protein from previously fried foods; therefore, restaurants should consider designating a fryer for one type of food. These recommendations for separate equipment, preparation and pick-up areas, and fryers might be difficult for many restaurants to implement, given resource and space limitations. Although research in this area is limited, one study found that conventional cleaning methods were effective in removing peanut proteins (*7*). Therefore, as an alternative in restaurants with limited resources or space, equipment and workspaces could be cleaned according to Food Code guidance (*5*) before preparing an allergen-free dish.

The findings in this report are subject to at least four limitations. First, because the interview responses were self-reported, they are subject to social desirability bias, which might have resulted in overreporting of appropriate practices. Second, because interviewed food workers and servers were selected by managers, and not at random, their responses might not represent the experiences or practices of all food workers and servers. Third, because the data were collected from English-speaking staff members only, they might not reflect practices in restaurants where no one speaks English. Finally, the low response rate (32.6%) might have resulted in an overrepresentation of restaurants with better food allergy practices.

Current recommendations for preventing allergic reactions in restaurants are based on actual cases of allergic reactions in restaurants, on expert opinion, and on research about how allergen proteins react. However, limited evaluation data currently exist on the effectiveness of the recommendations, and more research is needed on this topic. Food allergies are a serious food safety issue. Restaurants should ensure that all staff members are knowledgeable about food allergies, from preventing cross-contact to knowing how to respond in an emergency. Investing in and using dedicated equipment and designating areas for preparing allergen-free food can also reduce the risk for cross-contact. Increasing staff knowledge and awareness of food allergens can help restaurants better accommodate patrons with food allergies and increase the probability of a safe dining experience.

**Summary**

**What is already known about this topic?**

Food allergies affect an estimated 15 million persons in the United States and are responsible for approximately 30,000 emergency department visits and 150–200 deaths each year. Nearly half of fatal food allergy reactions over a 13-year period were caused by food from a restaurant or other food service establishment.

**What is added by this report?**

Fewer than half of members of the restaurant staffs surveyed in 278 restaurants had received training on food allergies. Topics frequently covered in the trainings were identifying the major food allergens, what to do if a customer has a food allergy, and how to prevent cross-contact of allergens. Although most restaurants have ingredient lists or recipes for at least some menu items, few have separate equipment or areas designated specifically for the preparation of allergen-free food.

**What are the implications for public health practice?**

It is important for restaurants to provide food allergy training for staff members and ensure that the training covers critical information. Restaurants can dedicate equipment and create separate areas that are specifically designated for preparing meals for customers with food allergies. Adopting these practices can reduce the risk for an allergic reaction among patrons.
